# Three serum metabolite signatures for diagnosing low-grade and high-grade bladder cancer

**DOI:** 10.1038/srep46176

**Published:** 2017-04-06

**Authors:** Guangguo Tan, Haibo Wang, Jianlin Yuan, Weijun Qin, Xin Dong, Hong Wu, Ping Meng

**Affiliations:** 1 School of Pharmacy, Fourth Military Medical University, Xi’an 710032, China; 2 Department of Urology, Xijing Hospital, Fourth Military Medical University, Xi’an 710032, China; 3 School of Pharmacy, Second Military Medical University, Shanghai, 200433, China

## Abstract

To address the shortcomings of cystoscopy and urine cytology for detecting and grading bladder cancer (BC), ultrahigh performance liquid chromatography (UHPLC) coupled with Q-TOF mass spectrometry in conjunction with univariate and multivariate statistical analyses was employed as an alternative method for the diagnosis of BC. A series of differential serum metabolites were further identified for low-grade(LG) and high-grade(HG) BC patients, suggesting metabolic dysfunction in malignant proliferation, immune escape, differentiation, apoptosis and invasion of cancer cells in BC patients. In total, three serum metabolites including inosine, acetyl-N-formyl-5-methoxykynurenamine and PS(O-18:0/0:0) were selected by binary logistic regression analysis, and receiver operating characteristic (ROC) test based on their combined use for HG BC showed that the area under the curve (AUC) was 0.961 in the discovery set and 0.950 in the validation set when compared to LG BC. Likewise, this composite biomarker panel can also differentiate LG BC from healthy controls with the AUC of 0.993 and 0.991 in the discovery and validation set, respectively. This finding suggested that this composite serum metabolite signature was a promising and less invasive classifier for probing and grading BC, which deserved to be further investigated in larger samples.

Bladder cancer (BC) is the second most common cancer of the genitourinary tract and a prevalent cause of cancer-related death worldwide^1^. BC is classified as low-grade (LG) and high-grade (HG) tumors based on the degree by which cancer cells histologically differ from normal bladder cells. LG BC has a low risk of recurrence and progression, whereas HG BC is frequently associated with tumor recurrence and progression to metastatic, lethal disease^2^. LG BC in general can be effectively treated with endoscopic local resection^3^. However, radical cystectomy are most commonly used to HG BC^4^. An early diagnosis of BC, especially discriminating HG BC from LG BC, could be of great importance in determining the appropriate treatment regimes.

Currently, the gold standard clinical method to diagnose BC is cystoscopy, although it is an invasive, unpleasant, and expensive approach. Sometimes it may miss a flat lesion, especially carcinoma *in situ* (CIS), which is considered HG BC. In addition, the voided-urine cytology approach is the most common for detection of HG BC; however, this method is subjective, costly, and it has interobserver variability as well as poor sensitivity and specificity, especially for LG BC^5^,^6^. Recently, many urine-based protein biomarkers were implicated in identification of HG BC. But until now none of the molecular markers have been generally accepted in the clinical practice^7^,^8^. Therefore, the identification of objective and noninvasive biomarkers that could discriminate HG BC from LG BC or healthy control would be of considerable clinical value in individualized treatment and improvement of prognosis for BC.

Evidence that cancer is primarily a metabolic disease enabled investigations to identify biomarkers for diagnosis and the pathological mechanism of many cancers from the perspective of metabolism^9^. Metabonomics focuses on the quantitative measurement of as many endogenous metabolites as possible in biosamples such as plasma and urine in order to acquire an overview of the metabolic or disease status^10^. It is known that a minor alteration at the level of gene or protein expression usually results in a significant change in small molecule metabolite level; therefore, metabonomics is an intensive and direct approach for capturing diseases specific metabolic signatures as possible biomarkers and obtaining fundamental mechanistic insights into carcinogenesis and staging of cancer^11^,^12^,^13^.

Previously, this method has been used to characterize the metabolic changes in the urine samples from BC patients^14^,^15^,^16^,^17^. To some extent, the levels of urinary metabolites are susceptible to the amount of liquid intake and the severe dietary influence (vegetarian or nonvegetarian)^18^,^19^. These intrinsic limitations make urine a less suitable biofluid to determine the differentiation of LG and HG BC. In contrast to urine, the overall metabolites changes in the serum of BC patients can be a better indicator of bladder dysfunction because serum is not only less prone to be affected by exogenous factors but also intra- and interindividual variations are far less^18^. To date, only one report has revealed serum metabolic variations between LG and HG BC using ^1^H NMR spectroscopy^20^. Given that ^1^H NMR analytical technology cannot provide complete coverage of the human metabolome due to the diverse physicochemical properties of metabolites and the relatively low sensitivity of ^1^H NMR, the determinations of the metabolite differentiations between LG and HG BC are still far from complete. It is meaningful to apply complementary metabonomic platforms such as mass spectrometry to identify these differentiations between LG and HG BC.

In this study, a non-targeted metabonomics platform based on ultrahigh performance liquid chromatography (UHPLC) coupled with Q-TOF mass spectrometry was employed to determine the global metabolic changes between healthy controls and BC patients, particularly focusing on the metabolic alterations between LG and HG BC. The objective of this study was to identify potential biomarkers of HG BC for early diagnosis and individualized treatment of BC and better understanding of the underlying mechanism of BC.

## Materials and Methods

### Chemicals and reagents

HPLC-grade Methanol and acetonitrile (ACN) were purchased from Merk (Darmstadt, Germany). Formic acid was obtained from Fluka (Buchs, Switzerland). 5-Methylcytidine, octanoylcarnitine, glycocholic acid and decanoylcarnitine were purchased from Sigma-Aldrich (St. Louis, MO). Phytosphingosine, sphinganine and palmitoylcarnitine were purchased from Acros Organics (NewJersey, USA). Lysophosphatidylcholine (20:0) and lysophosphatidylcholine (18:2) were purchased from Larodan AB (Malmo, Sweden). Hypoxanthine, inosine, kynurenine, hippuric acid, citric acid, indoleacetic acid and L-2-chlorophenylalanine (internal standard) were obtained from Shanghai Jingchun Reagent Co. Acetyl-N-formyl-5-methoxykynurenamine (AFMK) and indolelactic acid were purchased from Haorui Chemicals and Seebio Biotech (shanghai) Co.,Ltd, respectively. Ultrapure water was prepared with a Milli-Q water purification system (Millipore, Bedford, MA, USA).

### Recruitment and sample collection

The study protocol was approved by the Human Ethics Committee of the Fourth Military Medical University, and written informed consent was obtained from all study volunteers prior to participation. All procedures involving the human subjects were carried out in accordance with the recommendations of the Helsinki Declaration.

All the patients were first diagnosed by cystoscopy. Subsequently, transurethral resected tissue specimens were collected to conduct histopathological assessment to define LG and HG BC. Both stage and grade of tumors were determined according to the World Health Organization (WHO)/International Society of Urological Pathology (ISUP) classification criteria^21^. Adjacent, non-involved tissue samples were also gleaned from a few patients and used as controls. None had received chemotherapy or radiation before sample collection. Age- and sex-matched healthy controls (HC) were included. Exclusion criteria included renal pathology, urinary tract infections, diabetes, arthritis, any other malignancies, tuberculosis, endocrine disorders, drug abuse, and other conditions known to influence metabolic phenotype. A total of 172 serum samples were collected from 60 patients with LG BC, 60 patients with HG BC, and 52 HC in the morning before breakfast and stored at −80 °C until analysis.

### Histopathological Examinations

All tissue samples were fixed in 10% buffered formalin and embedded in paraffin wax for histopathology within 7 days. Tissues were sliced at a thickness of 5 to 6 μm using a microtome followed by staining with hematoxylin and eosin for pathological assessment by the department of pathology, Fourth Military Medical University. An average of 3–5 slices was examined for each tissue sample.

### Sample preparation

Fasting venous blood was obtained from all the above-mentioned individuals. The blood samples were allowed to clot for 90 min in freezer (4 °C) and then centrifuged at 3000 × g for 10 min. The supernatants (serum) were separated and transferred into new vials, and immediately stored frozen at −80 °C until UHPLC-Q-TOFMS analysis. Prior to the analysis, serum samples were thawed and vortexed for 5 s at room temperature. Subsequently, a 300 μL volume of methanol (containing 12.5 μg/ml L-2-chlorophenylalanine as the internal standard) was added to 100 μL of serum. After vigorous shaking for 1 min and incubation on ice for 10 min, the mixture was centrifuged at 14,000 × g for 15 min at 4 °C to precipitate the protein. All supernatant was transferred into an auto-sampler vial.

As part of the system conditioning and quality control (QC) process, 100 μL from each serum sample was pooled to generate a pooled QC sample and aliquots of 100 μL of this pooled sample were extracted by the same method. It was inserted through the analytical run at intervals of 9–15 real samples to be analyzed sixteen times. The QC samples were sufficiently spread out through the whole run as to ensure its validity.

### UHPLC-Q-TOFMS analysis

UHPLC analysis was performed on Agilent 1290 Infinity LC system (Agilent, Germany). Chromatographic separation was carried out at 40 °C on an ACQUITY UPLC HSS T3 C_18_ column (2.1 mm × 100 mm, 1.7 μm, Waters, Milford, MA). The column oven was set at 40 °C. The mobile phase consisted of 0.1% formic acid (A) and ACN modified with 0.1% formic acid (B), using a gradient elution of 5% B at 0–2 min, 5%–95% B at 2–13 min, 95% B at 13–15 min. The total run time was 20 min including equilibration. The flow rate was 400 μL/min and the injection volume was 3 μL.

An Agilent 6530 Accurate-Mass Quadrupole Time-of-Flight (Q-TOF) mass spectrometer (Agilent, USA) was used in the study. The Q-TOF mass spectrometer was operated in electrospray ionization source (ESI) positive ion mode with a capillary voltage of 3.5 kV, drying gas flow of 11 L/min, and a gas temperature of 350 °C. The nebulizer pressure was set at 45 psig. The fragmentor voltage was set at 120 V and skimmer voltage was set at 60 V. All analyses were acquired using a mixture of 10 mM purine (*m*/*z* 121.0508) and 2 mM hexakis phosphazine (*m*/*z* 922.0097) as internal standards to ensure mass accuracy and reproducibility. Data were collected in centroid mode and the mass range was set at *m*/*z* 50–1000 using extended dynamic range. Potential biomarkers were analyzed by MS/MS. MS spectra were collected at 2 spectra/s, and MS/MS spectra were collected at 0.5 spectra/s, with a medium isolation window (~4 *m/z*) and a fixed collision energy of 20 V. A negative ion scan was only employed when metabolite identification was carried out.

### Data handing and statistical analysis

The acquired UHPLC-Q-TOFMS data were exported in mzData format and then processed by XCMS package (http://metlin.scripps.edu/download/)^22^ as described in our previous publication^23^. The internal standard was used for data quality control (reproducibility) and data normalization. The ion peaks generated by the internal standard were also removed. The resulting three-dimensional matrix, including retention time and m/z pairs (variable indices), sample names (observations), and normalized ion intensities (variables), was exported for multivariate data analysis.

The normalized data was imported into a SIMCA-P (version 11.0, Umetrics, Umeå, Sweden) for principal component analysis (PCA) and partial least-squares discriminant analysis (PLS-DA) as well as orthogonal partial least-squares discriminant analysis (OPLS-DA) after mean-centering and unit variance (UV)-scaled for equal metabolite weighting. PCA makes it is possible to extract and display the systematic variation in the data. A PCA model provides a summary of all observations in the data table^24^. By relates a data matrix containing independent variables from samples (peak intensity values) to a matrix containing dependent variables (class belonging) for those samples, PLS-DA and OPLS-DA can remove those variations from the independent variables that are not correlated to the dependent variables, and enable reduced model complexity with preserved prediction ability^25^. Especially, the OPLS-DA can remove the uncorrelated signals resulting in information of the within-class variation^25^. The quality of the models was evaluated with the relevant R^2^X, R^2^Y, Q^2^Y, R^2^Y- intercepts and Q^2^Y-intercepts to avoid the risk of over-fitting^26^.

Differential metabolites were firstly selected according to the variable importance in the projection (VIP > 1) generated from the OPLS-DA model. Furthermore, a two-tailed Student’s t-test (p < 0.05) on the normalized peak areas was used to determine if different biomarker candidates obtained from OPLS-DA modeling were statistically significant between groups. The software MedCalc (version 11.4.2.0) was used to perform variable selection of potential biomarkers and receiver operating characteristic (ROC) analysis based on binary logistic regression model.

### Metabolite identification

Metabolite identification was carried out according to the authors’ previous work with slight modification^23^,^27^. Briefly, ions of interest were scanned in both positive and negative modes to facilitate the judgment of quasi-molecular ions. Potential molecular formulae were calculated by MassHunter Workstation Software-Qualitative Analysis (Agilent Technologies, California, United States). Structure information was obtained by searching freely accessible databases of HMDB (www.hmdb.ca)^28^, METLIN (http://metlin.scripps.edu)^29^ and KEGG (http://www.kegg.jp)^30^ utilizing detected molecular weights (under the above mentioned conditions, the mass difference was less than 10 ppm). At the same time, fragment ions were subjected to analysis through MS/MS to narrow the scope of target compounds. Finally, commercial standards were adopted to support the metabolites’ identification.

## Results

### Subject populations

The BC patients and healthy subjects were divided into a discovery set and a validation set. Serum metabolic profiling was performed on the two datasets. The discovery set was used to identify serum diagnostic markers for LG and HG BC; the validation set was used to independently validate the diagnostic performance of these biomarkers. A summary of patient demographics for the two datasets is presented in Table 1. Figure S1 exhibits typical UHPLC-Q-TOFMS total ion current (TIC) chromatographs, providing an overview of the metabolic profile in sera from HC, LG, and HG subjects. Histopathological examinations of the corresponding tissue samples are also shown alongside each TIC chromatographs. Normal urinary bladder epithelium is multilayered and is composed of basal, intermediate, and very large surface cells that look like an umbrella (Figure S1A). Low-grade BC exhibited fused and branching papillae. The cells were observed to be ordered and cohesive, with minimal crowding and minimal loss of polarity (Figure S1B). High-grade BC exhibited fused and branching papillae. The cells were observed to be disordered with frequent loss of polarity and discohesive (Figure S1C).

### Data quality assessment

A stability data set that displays a biochemical snapshot is very important to successful metabonomic study, reflecting the temporal state of an organism through its endogenous low molecular weight metabolites. To acquire reliable data, technical errors derived from sample collection, sample preparation, and UHPLC-Q-TOFMS analysis must be minimized to avoid confounding multivariate data analysis. In this study, samples from each group were alternated in random order. Moreover, a pooled QC sample was analyzed in parallel with the real samples to monitor the stability of the analytical system. PCA results of the QC sample demonstrated that the deviation of the peak areas was less than 2 SD (Figure S2), indicating that the data from the UHPLC-Q-TOFMS were statistically acceptable. In addition, the retention time deviation of the ions generated from XCMS package was less than 30 s for the UHPLC-Q-TOFMS analysis (data not shown), indicating high reproducibility. This confirms that differences observed between groups by multivariate statistical analysis were more likely to reflect varied metabolite profiles rather than analytical variation arising from technical errors.

### Identification of differential metabolites between LG/HG BC and healthy subjects

**Figure 1:**
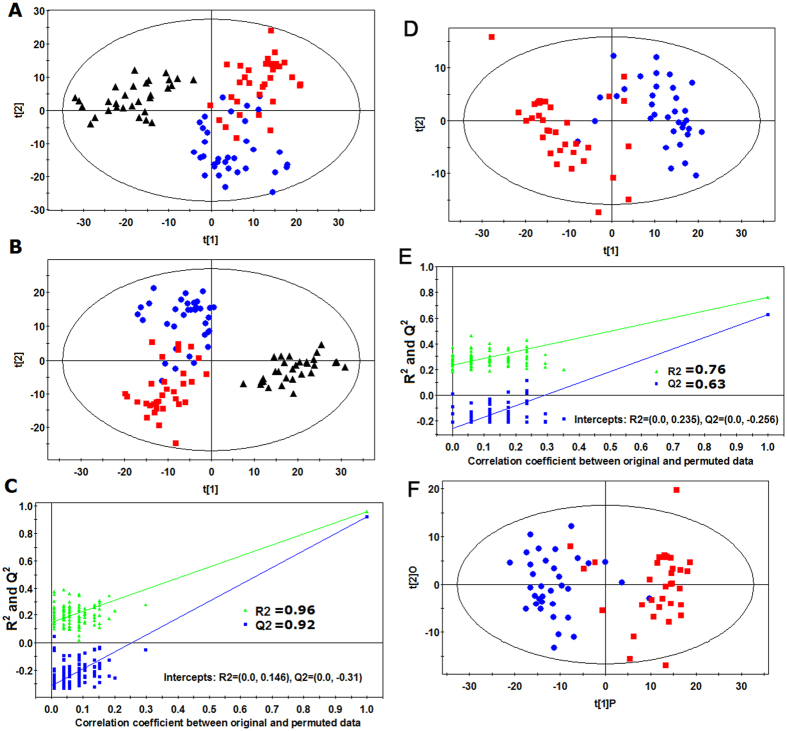
Multivariate data analysis based on the data from UHPLC-Q-TOFMS spectra of HG BC (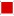), LG BC (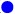), and HC (▲). (**A**) PCA score plot from HG BC, LG BC and HC, (**B**) PLS-DA score plot from HG BC, LG BC and HC, (**C**) Validation plot of PLS-DA model obtained using 99 permutation tests from HG BC, LG BC and HC, (**D**) PLS-DA score plot from HG BC and LG BC, (**E**) Validation plot of PLS-DA model obtained using 99 permutation tests from HG BC and LG BC, and (F) OPLS-DA score plot from HG BC and LG BC.

In this work, the BC patients were classified into two subgroups according to the histopathological evaluation of transurethral-resected tissue specimens, i.e., LG BC group and HG BC group. As an unsupervised multivariate statistical model, PCA was first performed to explore the metabolic differences among healthy subjects, LG BC patients and HG BC patients (12PCs, R^2^X = 0.882, Q^2^ = 0.532). A tendency in the PCA scores plot to separate BC patients and healthy subjects into the two classes was detected (Fig. 1A), indicating a significantly different serum metabolome between BC patients and healthy subjects. However, we did not observe an obvious difference between the LG BC and HG BC patients in the PCA scores plot. Furthermore, the supervised PLS-DA model was conducted (3PCs,R^2^Y = 0.816,Q^2^ = 0.773). A dramatic difference between BC patients and healthy subjects was observed in the PLS-DA score plot (Fig. 1B). Meanwhile, an obvious separation trend between LG BC and HG BC patients was also observed. Model validation with the number of permutations equalling 99 generated intercepts of R^2^ = 0.146 and Q^2^ = −0.310, which meant that the PLS-DA model was non-overfitting and reliable (Fig. 1C).

To identify the metabolites that can discriminate the metabolic distinctions, the supervised OPLS-DA models between the LG BC patients and healthy subjects and between HG BC patients and healthy subjects were performed. The OPLS-DA scores plots (Figure S3A (2PCs, R2Y = 0.990, Q2 = 0.959) and Figure S3C (2PCs, R2Y = 0.966, Q2 = 0.928)) displayed significant differences between the BC patients and healthy subjects. By combining the VIP values (>1) generated from OPLS-DA model with the results from the two-tailed Student’s t test, 25 and 25 metabolites were selected as differential metabolites for LG BC and HG BC patients, respectively (Table 2). Among these metabolites, 24 metabolites were the common characteristic of both LG BC and HG BC patients. 16 metabolites of them were unambiguously assigned by the comparison with the authentic standard compound. The structures and MS/MS spectra of the metabolites identified by data base searching are presented in Supporting Information Figure S4.

The serum samples of BC patients exhibited higher levels of 5-aminoimidazole ribonucleotide (AIR), 5-methylcytidine, hypoxanthine, inosine, kynurenine, AFMK, indolelactic acid, indoleacetic acid, glycocholic acid, PS(O-18:0/0:0), phytosphingosine, sphinganine, acylcarnitines and LysoPCs in combination with lower levels of citric acid, hippuric acid and LysoPE(22:6/0:0). These abnormal metabolite levels in serum reflected the alterations in the metabolic phenotype, which could provide insight into the underlying mechanism of BC.

### Identification of differential metabolites between LG BC and HG BC

To illustrate metabolite profiles between LG BC patients and HG BC patients and to identify potential biomarkers of HG BC, further analysis was performed to discriminate between LG BC and HG BC. The scores plot of PLS-DA (2PCs,R^2^Y = 0.763,Q^2^ = 0.627) showed a clear separation trend between LG BC and HG BC patients (Fig. 1D). Model validation with the number of permutations equalling 99 generated intercepts of R^2^ = 0.235 and Q^2^ = −0.250, which indicates that this PLS-DA model is not overfitting and reliable (Fig. 1E). The OPLS-DA model was further performed to discriminate LG BC patients and HG BC patients. A dramatic difference between the two groups of patients was observed (Fig. 1F), demonstrating that there existed the direct association between the metabolic profiles of serum and the histopathology of bladder tissue.

Similarly, 13 differential metabolites were identified as potential marker for discriminating between LG BC patients and HG BC patients. In contrast with the LG BC group, the level of AIR, hypoxanthine, inosine, AFMK, indoleacetic acid, glycocholic acid, PS(O-18:0/0:0), phytosphingosine, sphinganine, linolenyl carnitine and LysoPC(20:0) were increased in the HG BC group, whereas the levels of 3-hydroxydecanoyl carnitine and 3-hydroxyoctanoyl carnitine were markedly decreased. To quantitatively assess the capability of the potential marker metabolites to discriminate between LG BC patients and HG BC patients, the area under the ROC curve (AUC) and sensitivity as well as specificity were calculated individually for these 13 metabolites. The results are listed in Table 3, where AFMK and sphinganine demonstrated relatively high sensitivity (≥80%), and inosine, indoleacetic acid and PS(O-18:0/0:0) yielded specificity of more than 80%. However, none of the metabolites demonstrated both high sensitivity and specificity, making it necessary to apply multiple serum metabolites in the diagnosis of HG patients out of all BC patients. Complex diseases such as bladder cancer were generally involved with the disturbances of multiple metabolic pathways. Therefore, a panel of biomarkers will have more diagnostic power than one biomarker.

### Identification of a simplified HG BC metabolite signature

**Figure 2:**
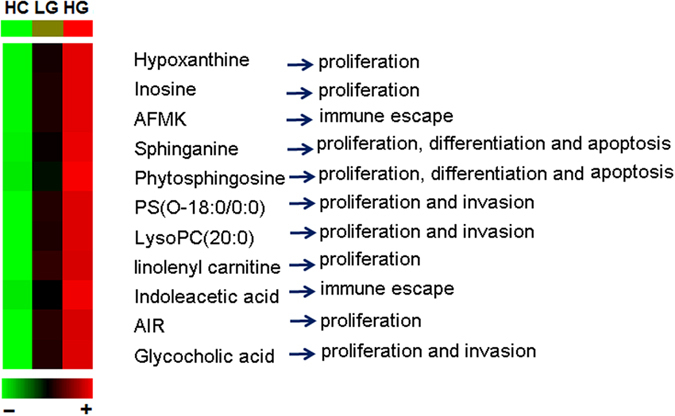
Heatmap and their potential function of 11 differential metabolites between LG BC and HG BC patients. It was generated by the average normalized peak areas. These metabolites showed progressive elevation with the progression of BC (from HC to LG BC to HG BC).

**Figure 3:**
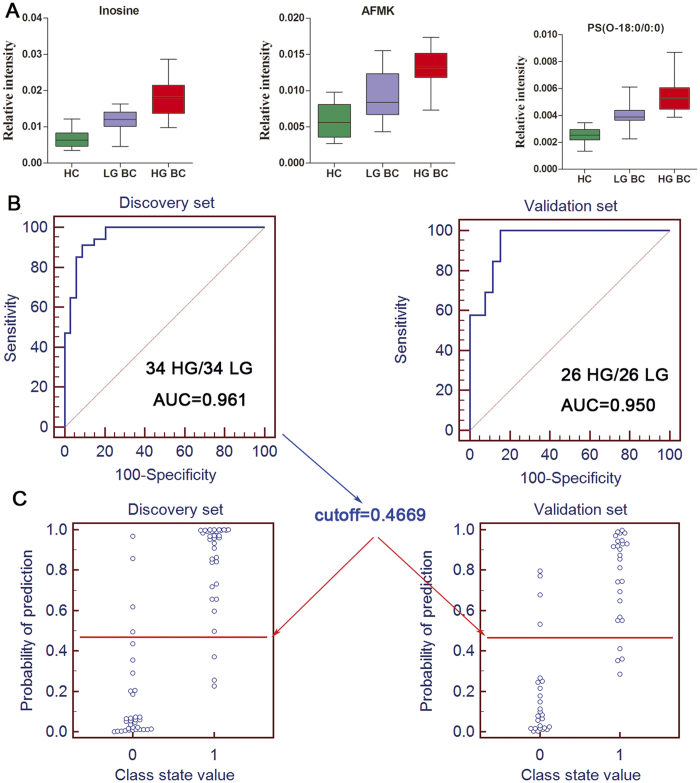
Box plots of serum inosine, AFMK and PS(O-18:0/0:0), ROC curves based on the binary logistic regression model by the combination of three serum metabolites from the HG and LG BC dataset and the prediction plots according to the optimal cutoff value obtained from ROC curves. (**A**) Box plots of the relative intensities of the three metabolites in HC, LG BC and HG BC. (**B**) The ROC curves of the discovery set (**B**, left) and validation set (**B**, right) were obtained from the established prediction model. (**C**) The optimal cutoff value was obtained (0.4669) and applied to evaluate the prediction capacity (89.7% for discovery set (C, left) and 84.6% for validation set (C, right)) of the current model, where 0 and 1 on the x axis represent LG BC and HG BC patients, respectively, and blue circle represent samples.

It was no doubt that a panel of biomarkers including these 13 serum metabolites will have more power to diagnose. However, diagnosis based on quantification of so many metabolites would not be convenient and economical in clinical practice. With bladder cancer progression (from healthy subjects to LG BC patients and then to HG BC patients), eleven metabolites demonstrated progressive increase trend (Fig. 2) and thus were further used as candidates. They could be assigned to different functions, such as proliferation, immune escape, differentiation, apoptosis and invasion, which will be discussed below. To identify a simplified serum metabolite signature that would be more practical in diagnosing HG BC, a binary logistic regression model with a stepwise optimization algorithm involving the 11 differential metabolites were subjected to variable selection based on the training set. As a result, three metabolites including inosine, AFMK and PS(O-18:0/0:0) were selected to establish a binary logistic regression model on the discovery set. The relative concentrations of these three serum metabolite biomarkers among the three groups are presented in Fig. 3A. The prediction model is as follows:


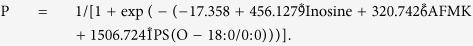


To evaluate the diagnostic performance of this prediction model with the simplified metabolite signature, ROC analyses on the prediction model was further conducted to obtain the diagnostic values of the biomarker panel. It demonstrated that a panel of three metabolites yielded an AUC of 0.961 (sensitivity is 88.2% and specificity is 91.2%; Fig. 3B, left). Based on this sensitivity and specificity of the ROC curves on the discovery set, an optimal cutoff value of 0.4669 was produced. According to this cutoff value, it was observed that 61 out of 68 samples (89.7%) could be accurately predicted in the discovery set (Fig. 3C, left), which indicated that LG BC and HG BC patients could be well-stratified with high accuracy by using the combination of inosine, AFMK and PS(O-18:0/0:0).

### Validation of the simplified HG BC biomarker panel

Based on the discovery set, a simplified HG BC metabolite signature was identified and preliminarily validated as an effective classifier of LG BC patients and HG BC patients. In order to validate this metabolite signature before proceeding to a larger-scale clinical trial, the simplified biomarker panel was used to classify blinded diverse samples from an independent test cohort of 26 LG BC patients and HG BC patients. ROC analysis yielded an AUC of 0.950 (84.6% sensitivity and 84.6% specificity; Fig. 3B, right) in discriminating HG BC patients from LG BC patients. Likewise, according to the cut off value (0.4669) from the training set, it was also found that 44 out of 52 samples (84.6%) in test set could be accurately predicted (Fig. 3C, right).

### Estimation and validation of this prediction model for LG BC

Similarly, when the LG BC patients were compared to healthy controls, the results indicated that a panel of the three metabolites based on this above prediction model generated an AUC of 0.993 with a sensitivity of 94.1% and a specificity of 93.3% and 0.991 with a sensitivity of 92.3% and a specificity of 90.9% for the discovery (Figure S5A, left) and validation sets (Figure S5A, right), respectively. Likewise, according to this sensitivity and specificity of the ROC curves on the discovery set, an optimal cutoff value of 0.0024 was obtained. Based on this cutoff value, it was found that 60 out of 64 samples (93.8%) in the discovery set (Figure S5B, left) as well as 44 out of 48 samples (91.7%) in validation set (Figure S6B, right) could be accurately predicted. This finding indicated that this simplified plasma metabolite signature was a “good” classifier of BC patients and healthy controls.

### Metabolic pathway analysis

**Figure 4:**
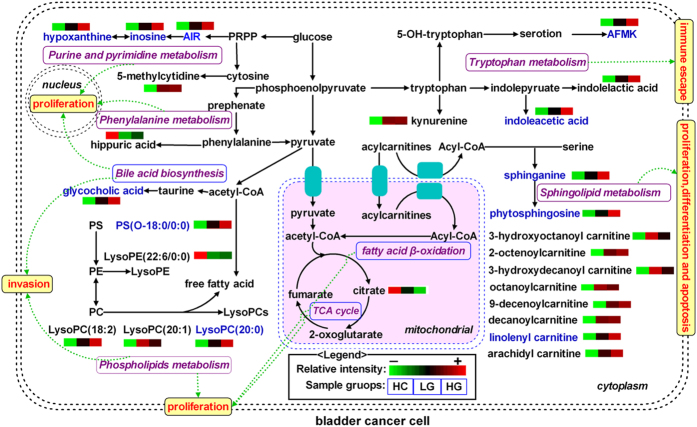
Proposed metabolic mechanisms associated with bladder cancer. The heatmap of the differential metabolites were generated by the average normalized peak areas, and metabolites in blue represent progressive increased trend from healthy control to LG BC patients to HG BC patient. Abbreviations: PRPP, phosphoribosyl pyrophosphate; PS, phosphatidylserine; PE, phosphoethanolamine; PC, phosphocholine; LysoPE, lysophosphatidylethanolamine; LysoPC, Lysophosphatidylcholine.

To explore the underlying molecular functions of these serum metabolite biomarkers, metabolic pathway analysis was performed. These metabolites were found to be primarily involved in purine, pyrimidine, tryptophan, phenylalanine, fatty acid β-oxidation, citrate cycle, phospholipid, sphingolipid, and bile acid biosynthesis metabolism. A simplified metabolic pathway is demonstrated in Fig. 4 based on a KEGG database online (http://www.genome.jp/kegg/pathway.html). We proposed potential metabolic dysfunction in BC, including malignant proliferation, immune escape, differentiation, apoptosis and invasion of BC cells.

## Discussion

Bladder cancer can be classified as low-grade and high-grade based on the degree by which cancer cells histologically differ from normal bladder cells, being high-grade BC more aggressive and invasive than low-grade^3^. Besides the precise detection of BC, the grading (LG or HG) of BC is crucial to determine the appropriate treatment regimes for BC; however, the consistency in pathological reports is a major issue^31^. Here, the possibility of using serum metabolite profiles of BC patients for indentifying the grade of BC at an early phase was preliminarily assessed. Serum-based metabolite profile could accurately discriminate HG BC, LG BC and healthy control cohorts, and different clusters of BC patients based on low and high grades were discovered in the OPLS-DA score plot of metabolic data, which indicated that disease status resulted in specific metabolic perturbations in the patients. Compared to the previous ^1^H NMR-based metabolomic study on LG and HG BC^20^, UHPLC-Q-TOFMS-based metabolomic approach provided larger coverage of LG and HG BC-related metabonome including sphingolipids, phospholipids, acylcarnitines and several metabolites of tryptophan pathway. Concentrating on the direct association of the serum metabolite profiles of BC and the histopathology of bladder tissue, a composite panel of potential biomarkers including inosine, AFMK and PS(O-18:0/0:0) was identified as a diagnostic tool, which could precisely distinguish not only between HG BC and LG BC but also between LG BC and healthy control. These findings demonstrated that this simplified metabolite signature should aid in the development of objective laboratory-based diagnostic tools for BC and the categorization of the LG and HG forms of BC.

It was found that the identified metabolites were primarily involved in purine, pyrimidine, tryptophan, phenylalanine, fatty acid β-oxidation, citrate cycle, phospholipid, sphingolipid, and bile acid biosynthesis metabolism. The disturbed metabolic pathways are discussed in detail below.

The levels of AIR, hypoxanthine and inosine were significantly increased with bladder cancer progression (from healthy subjects to LG BC patients and then to HG BC patients), which suggested that purine metabolism was upregulated in BC patients, especially in HG BC patients. AIR is an intermediate of purine nucleotide biosynthesis. In the normal breakdown of purine nucleotides, hypoxanthine and inosine were converted to uric acid^32^. However, the metabolic pathways were dysregulated as purine biosynthesis is favored due to enhanced cancer cells cycle activity, therefore AIR, hypoxanthine and inosine were accumulated for the *de novo* synthesis of purines. Likewise, due to the greater energetic state of the tumor cells, the level of 5-methylcytidine involved in RNA synthesis was elevated in BC patients, which suggested that pyrimidine metabolism was upregulated in BC patients.

The levels of kynurenine, AFMK, indolelactic acid and indoleacetic acid were increased in LG and HG BC patients compared to healthy controls, which suggested the perturbed tryptophan metabolism is implicated in BC patients. Although the potential role of the metabolic pathway in the development of BC has been reported in the previous urine and tissue metabonomic studies^33^,^34^, AFMK, indolelactic acid and indoleacetic acid were firstly identified as the diagnostic serum biomarkers of BC. Excessive tryptophan metabolites seem to play a role in suppressing antitumor immune responses, thus promoting cancer cells survival, through activation of aryl hydrocarbon receptor, which is involved in carcinogenesis^35^,^36^.

Hippuric acid is a metabolite of phenylalanine. In addition, phenylalanine, an important energy metabolism precursor, can also be transformed into some biomolecules, such as pyruvate, 2-oxoglutarate and fumarate, to enter into citrate cycle. Here, a significantly decreased level of hippuric acid was observed in BC subjects compared to healthy controls, suggesting the phenylalanine metabolism is perturbed in BC subjects. In agreement with this presumption, previous urine metabonomic studies have consistently reported that BC is associated with significantly reduced levels of metabolites in this metabolic pathway such as phenylalanine and hippuric acid^37^,^38^. One possible explanation was due to the fact that cancer cells require more energy for continuous growing and proliferation.

Acylcarnitine derivatives including 3-hydroxyoctanoyl carnitine, 2-octenoylcarnitine, 3-hydroxydecanoyl carnitine, octanoylcarnitine, 9-decenoylcarnitine, decanoylcarnitine, linolenyl carnitine, arachidyl carnitine were obviously increased in BC subjects relative to healthy controls. It is Acylcarnitines are essential for the transport of long chain fatty acids across the mitochondrial membrane for degradation and energy production, and that they have the ability to shuttle short chain fatty acids from the inside of the mitochondria to the cytosol^39^. During this process, carnitine is esterified to form acylcarnitine derivatives catalyzed by acetyl-CoA^40^. Therefore, the higher levels of acylcarnitines might be reflected the dysregulated fatty acid β-oxidation pathway in BC patients. In addition, citric acid, an important intermediate in citrate cycle, was observed to be reduced in BC subjects, which suggested that citrate cycle were impaired in BC patients. This result reflected the increased conversion of citrate into fatty acids necessary for β-oxidation to support the rapid proliferation of cancer cells. Numerous disorders have been described that lead to disturbances in energy production and intermediary metabolism, which are characterized by the abnormal levels of acylcarnitines and citrate^16^,^41^,^42^. This also implies a single metabolite will have limited use for an accurate diagnosis of BC, however, the biomarker panel would be more specificity.

The increased levels of LysoPC(18:2), LysoPC(20:1), LysoPC(20:0) and PS(O-18:0/0:0) and the decreased levels of LysoPE(22:6/0:0) were observed in BC subjects relative to healthy controls, suggesting that the perturbed phospholipid metabolism is implicated in BC. It is known that phospholipid metabolism regulate a variety of biological processes including cell proliferation, tumor cell invasion, and inflammation^43^,^44^. A possible explanation is due to the fact that proliferating tumor cells have a high demand of phospholipids for making up cell membranes, which they satisfy by overactivating the endogenous lipogenesis^45^.

The levels of phytosphingosine and sphinganine were significantly increased in BC subjects relative to healthy controls, which suggests that the perturbed sphingolipid metabolism is implicated in BC. Metabolism of sphingolipids was reported to have potential relation to cell growth, differentiation, apoptosis and angiogenesis^46^,^47^. Increased levels of phytosphingosine and sphinganine may reflect a relatively higher tumor cell proliferation rate and increased lipid membrane remodeling.

Gycocholic acid, a product of bile acid biosynthesis, is a hydrophobic conjugated bile acid. For the first time, we confirmed that gycocholic acid is up-regulated in BC subjects relative to healthy controls, suggesting that the perturbed bile acid biosynthesis is implicated in BC. A previous study has demonstrated that bladder cancer cell lines expressed the elevated peroxisome proliferator-activated receptors (PPARs) and liver X receptors (LXRs). These receptors could sense and metabolize bile acids^48^, which was in support of our data. In addition, much evidence has demonstrated that the hydrophobic conjugated bile acids are likely to be implicated in the aetiology of a number of different important cancers^49^, which could stimulate the growth and invasion of tumor cell^50^. Unfortunately, no more bile acids was identified in this study. Further investigations will be needed to search and elucidate other bile acids on a targeted metabonomics platform.

It should be noted that, among the identified biomarkers, indolelactic acid, indoleacetic acid and hippuric acid are gut bacteria-related metabolites^51^. It was possible that the changes in these metabolites were due to the gut microbiome changes in the BC patients. Further investigations will be needed to elucidate interactions between the gut microbiota and host metabolism in the BC patients, which may provide insight into the role of the gut microbiota in bladder cancer progression

## Conclusion

In conclusion, the present study revealed the UHPLC-Q-TOFMS based serum metabonomics approach is able to discover biomarkers applicable to the diagnosis of BC and the categorization of the LG and HG forms of BC. A panel of serum metabolite markers related with the transformation of bladder cancer pathology (LG or HG) was identified in this study, where the combination of serum inosine, AFMK and PS(O-18:0/0:0) could discriminate not only HG BC and LG BC but also LG BC and healthy control with satisfactory sensitivity as well as specificity. The results were on par with the gold-standard highly invasive and painful cystoscopic approach used in clinical practice. The elucidation of the correlation between serum metabolite profiles of BC patients and their histopathological status was potentially valuable both in aiding diagnosis and in providing novel insights regarding metabolism in BC as well as determining the appropriate treatment regimes of BC patients. In the future, analysis of additional large-scale samples should be explored to further validate the clinical utility of biomarkers described in this study.

## Additional Information

**How to cite this article**: Tan, G. *et al*. Three serum metabolite signatures for diagnosing low-grade and high-grade bladder cancer. *Sci. Rep.* **7**, 46176; doi: 10.1038/srep46176 (2017).

**Publisher's note:** Springer Nature remains neutral with regard to jurisdictional claims in published maps and institutional affiliations.

## Supplementary Material

Supplementary Information

## Figures and Tables

**Table 1:** Demographic and clinical details of recruited subjects.

Parameters	Discovery set (Race: Chinese)			Validation set (Race: Chinese)		
	LGBC	HGBC	Healthy	LGBC	HGBC	Healthy
Sample size	34	34	30	26	26	22
Gender (male/female)	29/5	30/4	24/5	22/4	21/5	17/4
BMI (median, range)	23.1 (17.1–27.6)	22.3 (16.6–28.2)	23.5 (17.5–27.7)	23.5 (16.7–28.2)	22.7 (16.3–27.4)	23.6 (17.0–28.4)
Hematuria	4 (11.7%)	5 (14.7%)	0	3 (11.5%)	3 (11.5%)	0
Medications	0	0	0	0	0	0
BC stage						
Ta	2 (5.9%)	2 (5.9%)	—	1 (3.8%)	2 (7.7%)	—
T1	19 (55.9%)	6 (17.67%)	—	15 (57.7%)	4 (15.3)	—
T2	13 (38.2%)	17 (50.0%)	—	10 (38.5%)	13 (50.0%)	—
T3	—	7 (20.6%)	—	—	5 (19.3%)	—
T4	—	2 (5.9%)	—	—	2 (7.7%)	—
Smoking habit						
Smokers	23 (67.6%)	21 (61.8%)	22 (73.3%)	17 (65.4%)	16 (61.5%)	15 (68.2%)
Ex-smokers	3 (8.3%)	5 (14.7%)	0	2 (7.7%)	2 (7.7%)	0
Nonsmokers	8 (23.5%)	8 (23.5%)	8 (26.7%)	7 (26.9%)	8 (30.8%)	7 (31.8%)

**Table 2:** Differential serum metabolites among LG BC patients and HG BC patients as well as healthy controls.

No.	t_R_ (min)	m/z^a^	Formula	Metabolites^*b*^	LGBC/healthy			HGBC/healthy		
					VIP^e^	*p* value^f^	FC^g^	VIP	*p* value	FC
1	0.66	296.0661	C_8_H_14_N_3_O_7_P	*5-Aminoimidazole ribonucleotide*^*c*^	1.52	2.04 × 10^−10^	1.63	1.81	9.38 × 10^−24^	2.19
2	0.70	258.1050	C_10_H_15_N_3_O_5_	*5-Methylcytidine*^*d*^	1.64	1.51 × 10^−12^	1.48	1.44	2.03 × 10^−11^	1.51
3	1.02	137.0457	C_5_H_4_N_4_O	*Hypoxanthine*^*d*^	1.23	1.15 × 10^−6^	1.45	1.54	7.02 × 10^−13^	1.96
4	1.05	215.0160*	C_6_H_8_O_7_	*Citric acid*^*d*^	1.57	9.64 × 10^−6^	0.72	1.16	7.04 × 10^−12^	0.57
5	1.44	269.0880	C_10_H_12_N_4_O_5_	*Inosine*^*d*^	1.60	8.66 × 10^−12^	1.79	1.67	3.48 × 10^−17^	2.72
6	2.24	209.0917	C_10_H_12_N_2_O_3_	*Kynurenine*^*d*^	1.83	1.59 × 10^−17^	1.61	1.73	1.02 × 10^−18^	1.64
7	4.82	180.0651	C_9_H_9_NO_3_	Hippuric acid^*d*^	1.10	2.45 × 10^−5^	0.68	0.94	8.07 × 10^−4^	0.74
8	4.83	265.1182	C_13_H_16_N_2_O_4_	*Acetyl-N-formyl-5-methoxykynurenamine*^*d*^	1.15	8.24 × 10^−6^	1.61	1.78	1.62 × 10^−18^	2.28
9	5.67	304.2112	C_15_H_29_NO_5_	*3-hydroxyoctanoyl carnitine*^*c*^	1.48	9.93 × 10^−10^	2.13	1.43	3.03 × 10^−9^	1.57
10	6.01	206.0806	C_11_H_11_NO_3_	*Indolelactic acid*^*d*^	1.49	5.25 × 10^−10^	1.60	1.78	6.37 × 10^−18^	2.14
11	6.34	286.2011	C_15_H_27_NO_4_	*2-Octenoylcarnitine*^*c*^	1.43	5.62 × 10^−9^	1.74	1.49	9.10 × 10^−12^	1.91
12	6.63	176.0697	C_10_H_9_NO_2_	*Indoleacetic acid*^*d*^	1.41	1.06 × 10^−8^	1.58	1.69	3.15 × 10^−17^	2.54
13	6.91	332.2428	C_17_H_33_NO_5_	*3-hydroxydecanoyl carnitine*^*c*^	1.85	1.62 × 10^−18^	2.18	1.47	1.45 × 10^−10^	1.67
14	6.93	288.2168	C_15_H_29_NO_4_	*Octanoylcarnitine*^*d*^	1.53	1.55 × 10^−10^	1.48	1.04	2.98 × 10^−5^	1.51
15	7.58	314.2325	C_17_H_31_NO_4_	*9-Decenoylcarnitine*^*c*^	1.79	3.06 × 10^−16^	1.66	1.50	7.72 × 10^−11^	1.80
16	7.80	466.3159	C_26_H_43_NO_6_	*Glycocholic acid*^*d*^	1.02	1.01 × 10^−4^	1.68	1.60	1.79 × 10^−13^	2.34
17	7.97	316.2482	C_17_H_33_NO_4_	*Decanoylcarnitine*^*d*^	1.47	1.67 × 10^−9^	1.54	1.07	2.27 × 10^−5^	1.57
18	8.09	512.3345	C_24_H_50_NO_8_P	*PS(O-18:0/0:0)*^*c*^	1.62	2.78 × 10^−12^	1.59	1.74	8.93 × 10^−20^	2.15
19	8.39	318.3003	C_18_H_39_NO_3_	Phytosphingosine^*d*^	0.98	1.58 × 10^−4^	1.33	1.51	8.99 × 10^−13^	1.87
20	9.23	302.3052	C_18_H_39_NO_2_	*Sphinganine*^*d*^	1.49	8.04 × 10^−10^	1.44	1.77	2.67 × 10^−20^	1.98
21	9.85	422.3263	C_25_H_43_NO_4_	*linolenyl carnitine*^*c*^	1.79	2.60 × 10^−16^	1.81	1.62	2.14 × 10^−15^	2.51
22	10.26	526.2934	C_27_H_44_NO_7_P	*LysoPE(22:6/0:0)*^*c*^	1.37	3.91 × 10^−8^	0.67	1.29	4.85 × 10^−8^	0.69
23	10.27	520.3406	C_26_H_50_NO_7_P	*LysoPC(18:2)*^*d*^	1.61	5.18 × 10^−12^	1.34	1.74	2.82 × 10^−20^	1.61
24	12.18	456.4043	C_27_H_53_NO_4_	*Arachidyl carnitine*^*c*^	1.56	4.47 × 10^−11^	1.35	1.61	5.23 × 10^−14^	1.53
25	12.23	550.3860	C_28_H_56_NO_7_P	*LysoPC(20:1)*^*c*^	1.79	2.64 × 10^−16^	1.78	1.61	1.77 × 10^−13^	1.51
26	13.44	552.4028	C_28_H_58_NO_7_P	*LysoPC(20:0)*^*d*^	1.41	1.07 × 10^−8^	1.53	1.60	6.09 × 10^−15^	2.05

^a^The ion marked were “*“ were [M + Na]^+^ and other ion were [M + H]^+^. ^b^The metabolites marked with “c” were putatively annotated, the metabolites marked with “d” were structurally identified by reference standards, and those in italic type were identified as differential metabolites that are common to both LG BC/HC and HG BC/HC. ^e^Variable importance in the projection (VIP) was obtained from the OPLS-DA model. ^f^The p value was calculated from Student’s t test. ^g^Fold change was calculated from the normalized peak area between LG BC group vs HC group or between HG BC group vs HC group.

**Table 3:** Differential metabolites for discrimination between HG BC patients and LG BC patients.

No.	t_R_(min)	m/z^*a*^	Formula	Metabolites^*b*^	VIP^*e*^	*p* value^*f*^	FC^*g*^	AUC^*h*^	sensitivity (%)	specificity (%)
1	0.66	296.0661	C_8_H_14_N_3_O_7_P	*5-Aminoimidazole ribonucleotide*^*c*^	1.71	1.79 × 10^−8^	1.34	0.79(0.68–0.89)	0.73	0.68
2	1.02	137.0457	C_5_H_4_N_4_O	*Hypoxanthine*^*d*^	1.49	2.04 × 10^−6^	1.35	0.79(0.67–0.88)	0.68	0.70
3	1.44	269.0880	C_10_H_12_N_4_O_5_	*Inosine*^*d*^	1.72	1.34 × 10^−8^	1.44	0.86(0.75–0.93)	0.68	0.82
4	4.83	265.1182	C_13_H_16_N_2_O_4_	*Acetyl-N-formyl-5-methoxykynurenamine*^*d*^	1.54	8.81 × 10^−7^	1.41	0.80(0.69–0.89)	0.82	0.70
5	5.67	304.2112	C_15_H_29_NO_5_	3-hydroxyoctanoyl carnitine^*c*^	1.08	9.55 × 10^−4^	0.73	0.67(0.55–0.78)	0.70	0.65
6	6.63	176.0697	C_10_H_9_NO_2_	*Indoleacetic acid*^*d*^	1.82	1.20 × 10^−9^	1.34	0.88(0.77–0.94)	0.76	0.80
7	6.91	332.2428	C_17_H_33_NO_5_	3-hydroxydecanoyl carnitine^*c*^	1.38	1.39 × 10^−5^	0.76	0.80(0.69–0.89)	0.76	0.73
8	7.80	466.3159	C_26_H_43_NO_6_	*Glycocholic acid*^*d*^	1.19	2.64 × 10^−4^	1.39	0.77(0.65–0.86)	0.62	0.70
9	8.09	512.3345	C_24_H_50_NO_8_P	*PS(O-18:0/0:0)*^*c*^	1.69	2.78 × 10^−8^	1.35	0.88(0.77–0.94)	0.73	0.82
10	8.39	318.3003	C_18_H_39_NO_3_	*Phytosphingosine*^*d*^	1.60	2.40 × 10^−7^	1.40	0.84(0–.73–0.92)	0.76	0.68
11	9.23	302.3052	C_18_H_39_NO_2_	*Sphinganine*^*d*^	1.88	1.60 × 10^−10^	1.38	0.91(0.81–0.96)	0.80	0.76
12	9.85	422.3263	C_23_H_45_NO_4_	*linolenyl carnitine*^*c*^	1.42	8.24 × 10^−6^	1.38	0.77(0.65–0.86)	0.68	0.76
13	13.44	552.4028	C_28_H_58_NO_7_P	*LysoPC(20:0)*^*d*^	1.39	1.22 × 10^−5^	1.34	0.76(0.64–0.86)	0.70	0.65

^*a*^The ions were [M + H]^+^. ^*b*^The metabolites marked with “c” were putatively annotated, the metabolites marked with “d” were structurally identified by reference standards, and those in italic type were subjected to variable selection analysis prior to a binary logistic regression analysis. ^*e*^Variable importance in the projection (VIP) was obtained from the OPLS-DA model. ^*f*^The p value was calculated from Student’s t test. ^g^Fold change was calculated from the normalized peak area of HG BC group vs LG BC group. ^*h*^Area under the receiver operating characteristic (ROC) curve, with the 95% confidence interval (CI) range in parentheses.
