# Supplementary material for: Three serum metabolite signatures for diagnosing low-grade and high-grade bladder cancer
Source: Sci Rep. 2017 Apr 6;7:46176. doi: 10.1038/srep46176 (PMC5382774; doi:10.1038/srep46176)
Supplement: Supplementary Information [file srep46176-s1.doc]

# Supplementary data

**Three serum metabolite signatures for diagnosing low-grade and high-grade bladder cancer**

Guangguo Tan^1#^, Haibo Wang^1#^, Jianlin Yuan^2^, Weijun Qin^2^, Xin Dong^3^, Hong Wu^1*^, Ping Meng^2*^

^1.^School of Pharmacy, Fourth Military Medical University, Xi’an 710032, China

^2.^Department of Urology, Xijing Hospital, Fourth Military Medical University, Xi’an 710032, China

^3^. School of Pharmacy, Second Military Medical University, Shanghai, 200433, China

^#^Authors with equal contribution to the research.

Corresponding Author: E-mail: mengpingfmmu@126.com; Tel.: +86-29-8477-5321; Fax: +86-29-8477-5321 (Ping Meng). E-mail: wuhong@fmmu.edu.cn, Tel.: +86-29-8477-6823 (Hong Wu)

# Supplementary data

**Figure S1**. Typical TICs obtained from plasma sample in different group in ESI positive mode based on UHPLC-Q-TOFMS. (A) Healthy control, (B) low-grade bladder cancer and (C) high-grade bladder cancer.

**Figure S2**. Quality control (QC) plots of sixteen repeated runs of UHPLC–Q-TOFMS analysis using an artificial sample generated by principle component analysis using component 1 and 2. Peak area deviation could be evaluated by distribution of the runs. X-axis: run order; Y-axis: standard deviation. (A) QC plot for the first component from UHPLC–Q-TOFMS data; (B) QC plot for the second component from UHPLC–Q-TOFMS data;

**Figure S3.** Scores plots of OPLS-DA of LG BC or HG BC patients vs healthy controls, and permutation tests of their corresponding PLS-DA models. (A). scores plot of OPLS-DA of LG BC patients (●) versus healthy controls (▲), (B). permutation test of LG BC patients versus healthy controls, (C). scores plot of OPLS-DA of HG BC patients (■)versus healthy controls (▲), (D). permutation test of HG BC patients versus healthy controls.

**Figure S4**. Structures and MS/MS spectra of the metabolites. (A) 5-Aminoimidazole ribonucleotide, (B) 5-Methylcytidine, (C) Hypoxanthine (D) Citric acid, (E) Inosine, (F) Kynurenine, (G) Hippuric acid, (H) Acetyl-N-formyl-5-methoxykynurenamine, (I) 3-hydroxyoctanoyl carnitine (G) Indolelactic acid, (K) 2-Octenoylcarnitine, (L) Indoleacetic acid, (M) 3-hydroxydecanoyl carnitine, (N) Octanoylcarnitine, (O) 9-Decenoylcarnitine, (P) Glycocholic acid, (Q) Decanoylcarnitine, (R) PS(O-18:0/0:0), (S) Phytosphingosine, (T) Sphinganine, (U) linolenyl carnitine, (V) LysoPE(22:6/0:0), (W) LysoPC(18:2), (X) Arachidyl carnitine, (Y) LysoPC(20:1), and (Z) LysoPC(20:0).

**Figure S5**. ROC curves based on the binary logistic regression model by the combination of three serum metabolites (inosine, AFMK and PS(O-18:0/0:0) from the HC and LG BC dataset and the prediction plots according to the optimal cutoff value obtained from ROC curves. (A) The ROC curves of the discovery set (A, left) and validation set (A, right) were obtained from the established prediction model. (B) The optimal cutoff value was obtained (0.0024) and applied to evaluate the prediction capacity (93.8% for discovery set (B, left) and 91.7% for validation set (B, right) ) of the current model, where 0 and 1 on the x axis represent healthy controls and LG BC patients, respectively, and blue circle represent samples.


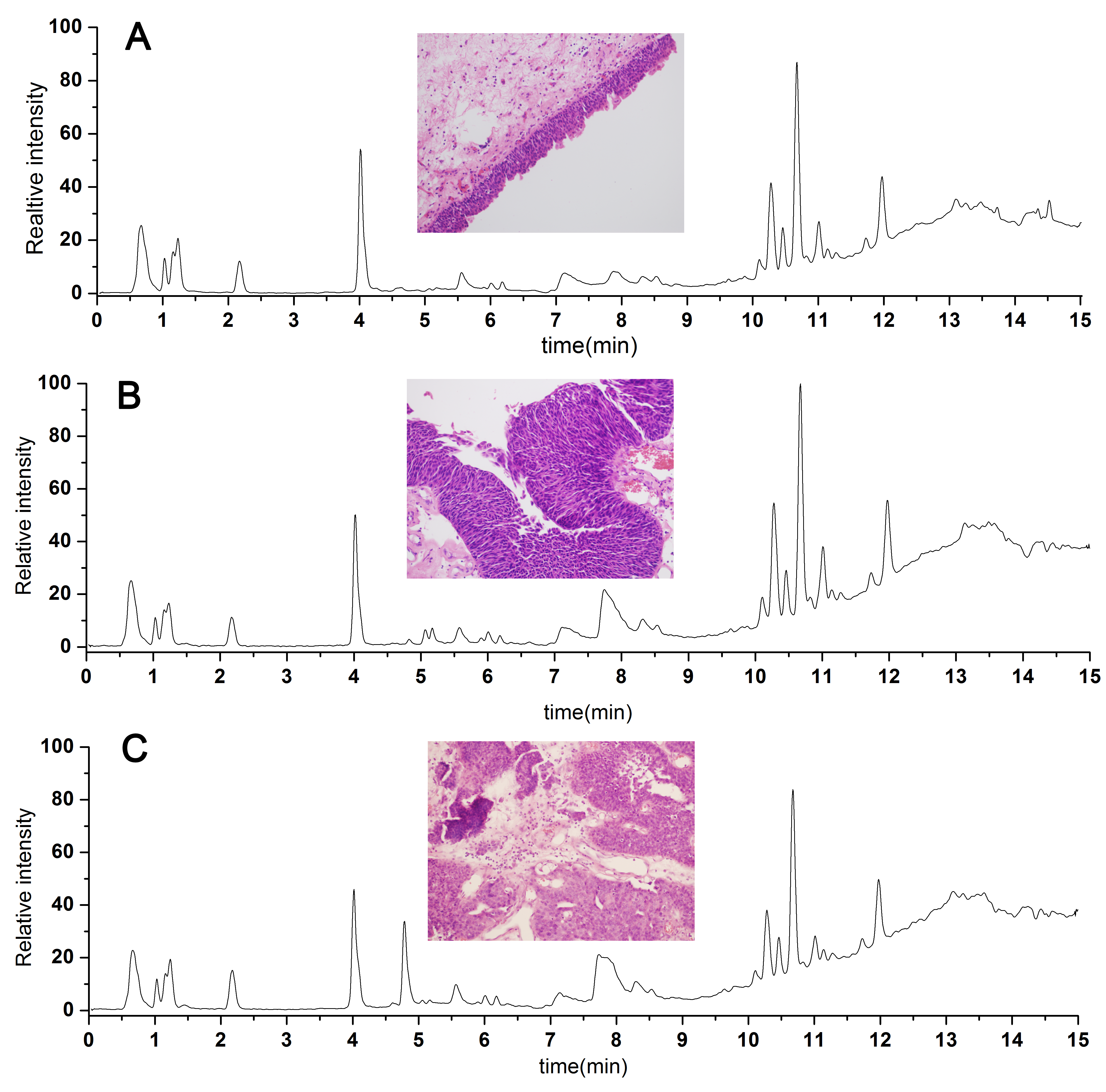


**Figure S1**. Typical TICs obtained from plasma sample in different group in ESI positive mode based on UHPLC-Q-TOFMS. (A) Healthy control, (B) low-grade bladder cancer and (C) high-grade bladder cancer.


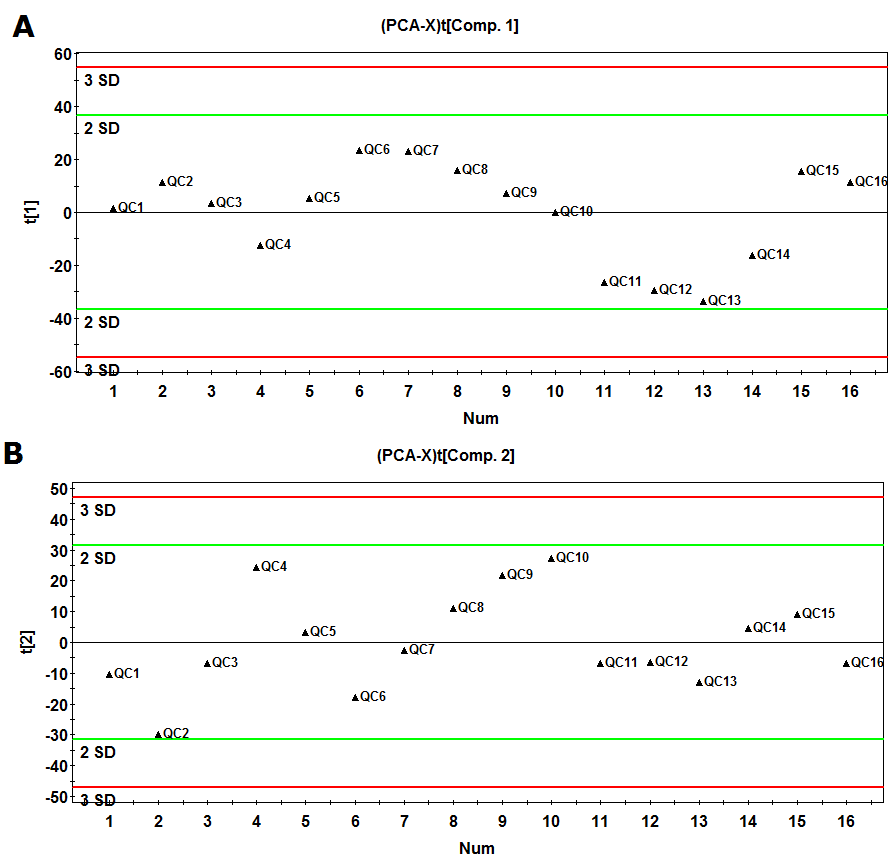


**Figure S2**. Quality control (QC) plots of sixteen repeated runs of UHPLC–Q-TOFMS analysis using an artificial sample generated by principle component analysis using component 1 and 2. Peak area deviation could be evaluated by distribution of the runs. X-axis: run order; Y-axis: standard deviation. (A) QC plot for the first component from UHPLC–Q-TOFMS data; (B) QC plot for the second component from UHPLC–Q-TOFMS data;


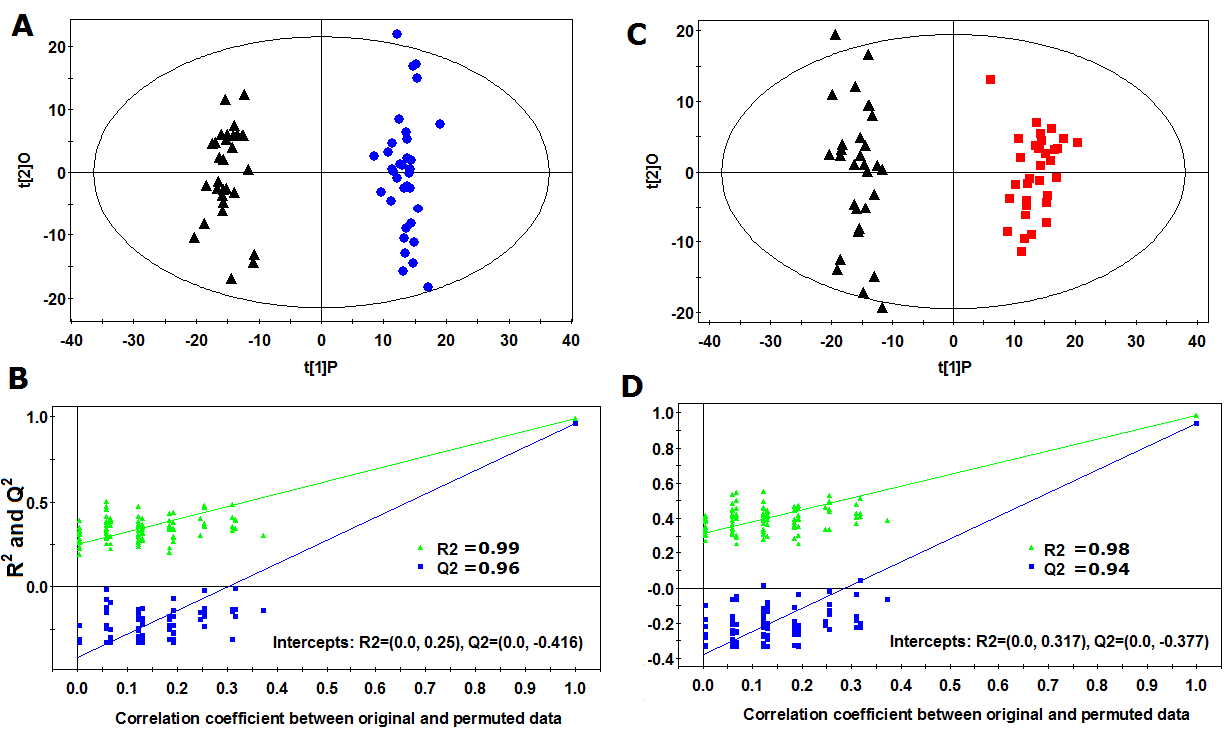


**Figure S3.** Scores plots of OPLS-DA of LG BC or HG BC patients vs healthy controls, and permutation tests of their corresponding PLS-DA models. (A). scores plot of OPLS-DA of LG BC patients (●) versus healthy controls (▲), (B). permutation test of LG BC patients versus healthy controls, (C). scores plot of OPLS-DA of HG BC patients (■)versus healthy controls (▲), (D). permutation test of HG BC patients versus healthy controls.


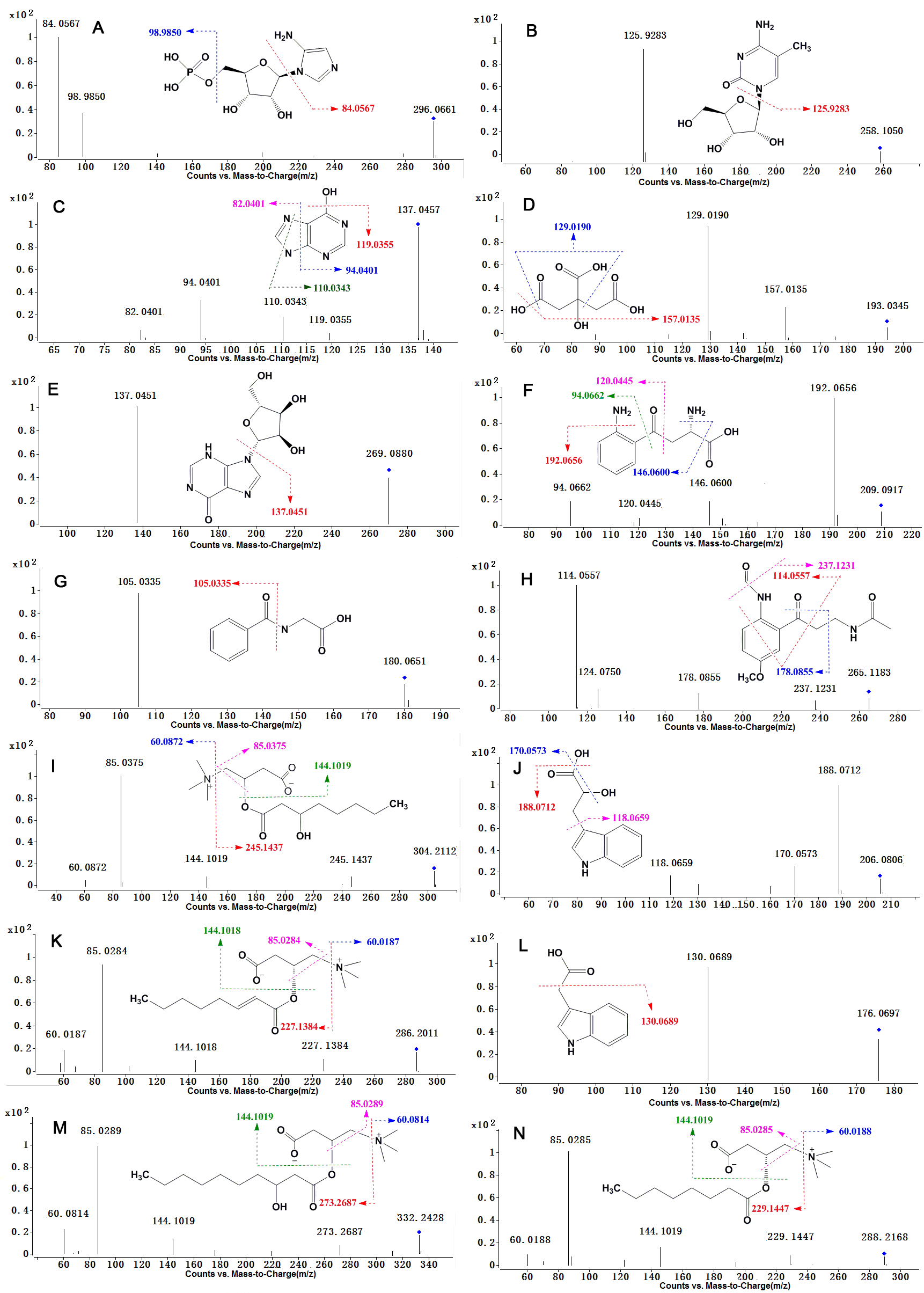


**Figure S4**. Structures and MS/MS spectra of the metabolites. (A) 5-Aminoimidazole ribonucleotide, (B) 5-Methylcytidine, (C) Hypoxanthine (D) Citric acid, (E) Inosine, (F) Kynurenine, (G) Hippuric acid, (H) Acetyl-N-formyl-5-methoxykynurenamine, (I) 3-hydroxyoctanoyl carnitine (G) Indolelactic acid, (K) 2-Octenoylcarnitine, (L) Indoleacetic acid, (M) 3-hydroxydecanoyl carnitine, (N) Octanoylcarnitine, (O) 9-Decenoylcarnitine, (P) Glycocholic acid, (Q) Decanoylcarnitine, (R) PS(O-18:0/0:0), (S) Phytosphingosine, (T) Sphinganine, (U) linolenyl carnitine, (V) LysoPE(22:6/0:0), (W) LysoPC(18:2), (X) Arachidyl carnitine, (Y) LysoPC(20:1), and (Z) LysoPC(20:0).


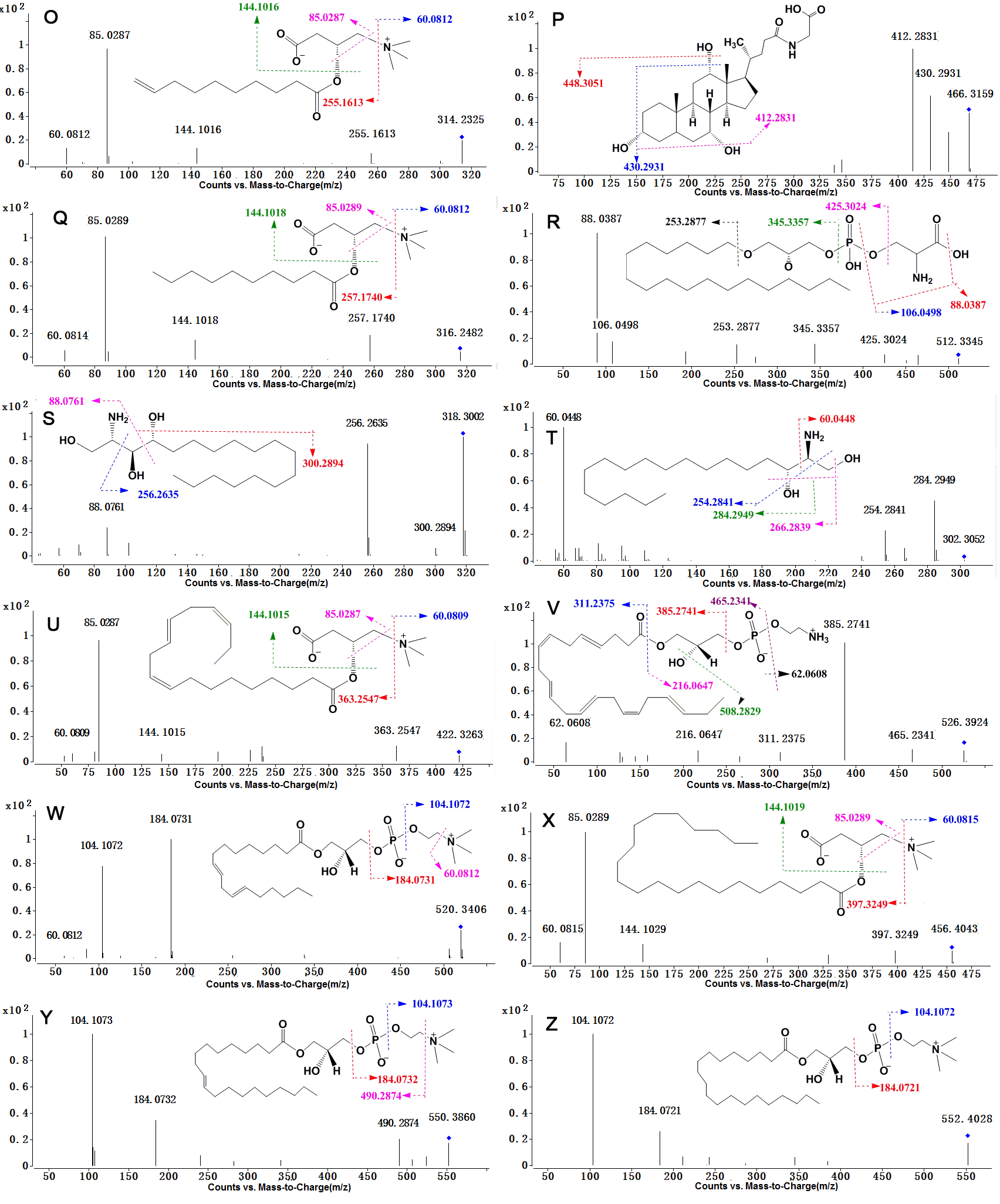


**Figure S4** continued


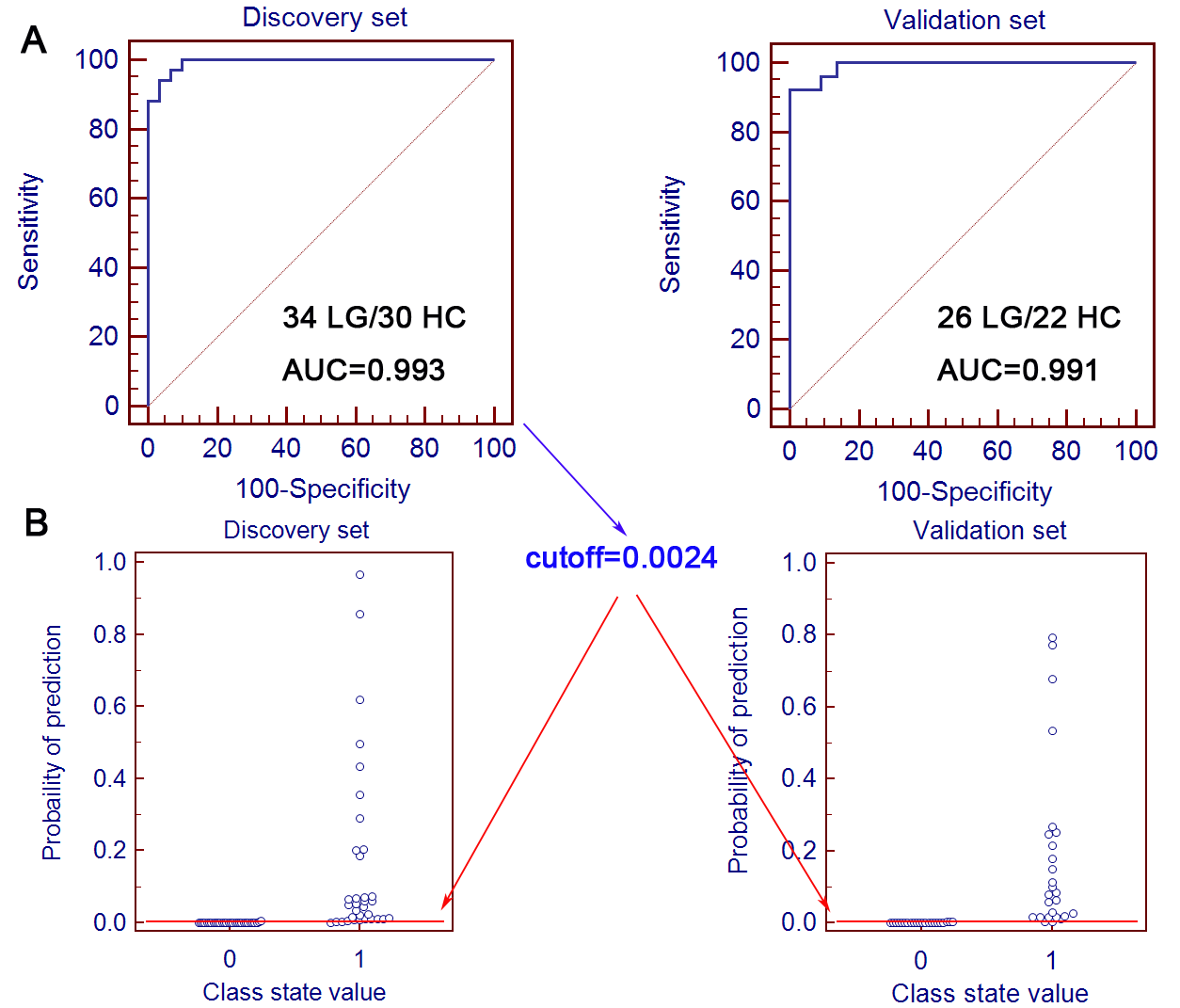


**Figure S5**. ROC curves based on the binary logistic regression model by the combination of three serum metabolites (inosine, AFMK and PS(O-18:0/0:0) from the HC and LG BC dataset and the prediction plots according to the optimal cutoff value obtained from ROC curves. (A) The ROC curves of the discovery set (A, left) and validation set (A, right) were obtained from the established prediction model. (B) The optimal cutoff value was obtained (0.0024) and applied to evaluate the prediction capacity (93.8% for discovery set (B, left) and 91.7% for validation set (B, right) ) of the current model, where 0 and 1 on the x axis represent healthy controls and LG BC patients, respectively, and blue circle represent samples.
